# Mining Temporal Dynamics With Support Vector Machine for Predicting the Neural Fate of Target in Attentional Blink

**DOI:** 10.3389/fnsys.2021.734660

**Published:** 2021-10-29

**Authors:** Yuan Yao, Yunying Wu, Tianyong Xu, Feiyan Chen

**Affiliations:** ^1^Bio-X Laboratory, Department of Physics, Zhejiang University, Hangzhou, China; ^2^Department of Education, Suzhou University of Science and Technology, Suzhou, China; ^3^Institute of Psychological Sciences, Hangzhou Normal University, Hangzhou, China; ^4^Center for Cognition and Brain Disorders, The Affiliated Hospital of Hangzhou Normal University, Hangzhou, China; ^5^Zhejiang Key Laboratory for Research in Assessment of Cognitive Impairments, Hangzhou, China

**Keywords:** attentional blink, EEG, phase locking value (PLV), support vector machine (SVM), top-down process

## Abstract

Our brains do not mechanically process incoming stimuli; in contrast, the physiological state of the brain preceding stimuli has substantial consequences for subsequent behavior and neural processing. Although previous studies have acknowledged the importance of this top-down process, it was only recently that a growing interest was gained in exploring the underlying neural mechanism quantitatively. By utilizing the attentional blink (AB) effect, this study is aimed to identify the neural mechanism of brain states preceding T2 and predict its behavioral performance. Interarea phase synchronization and its role in prediction were explored using the phase-locking value and support vector machine classifiers. Our results showed that the phase coupling in alpha and beta frequency bands pre-T1 and during the T1–T2 interval could predict the detection of T2 in lag 3 with high accuracy. These findings indicated the important role of brain state before stimuli appear in predicting the behavioral performance in AB, thus, supporting the attention control theories.

## Introduction

Our brains do not process incoming stimuli passively; rather, if a certain stimulus is to be perceived, it depends in part on the current state of the brain (Hanslmayr et al., 2011). In other words, whether a stimulus is perceived as the result of the interaction of the bottom-up and top-down processing approaches. Most of the previous cognitive science research focuses on the process after the stimulus appears. For instance, consciously reported target stimuli to display a stronger and more sustained pattern of activation (Marti and Dehaene, 2017). Late components P3 showed a significant non-linear increase in amplitude between seen and unseen conditions (Del Cul et al., 2007). However, top-down predictions shape how we perceive and comprehend the world has become increasingly influential in the field of systems neuroscience (Teufel and Fletcher, 2020). For instance, prestimulus alpha power has been proved to be a neural predictor of visual awareness (Benwell et al., 2021). Synchronous oscillations in the alpha frequency band inhibit the perception of shortly presented stimuli whereas synchrony in higher frequency ranges (>20 Hz) enhances visual perception, indicating the attentional state could predict perception performance on a single trial basis (Hanslmayr et al., 2007). Besides, the early transient global increase of phase synchrony in the gamma frequency range has also been reported to mediate the access to conscious perception (Melloni et al., 2007). Therefore, identifying the neural mechanism of pre-stimulus brain states and predicting whether or not a sensory stimulus will be perceived are two key goals in modern cognitive neuroscience (Hanslmayr et al., 2011).

To contrast between stimuli accessing consciousness and those that do not, previous researchers have developed a broad variety of paradigms (Kim and Blake, 2005). Here, a basic distinction must be clarified before we adopt a certain kind of paradigm, that is, whether the non-conscious stimulus is subliminal or preconscious (Dehaene et al., 2006; Kanai et al., 2010). According to Dehaene and Changeux (2011), subliminal stimuli refer to a kind of bottom-up, stimulus-driven information, even in the case of concentrated attention, so that it cannot be detected. A preconscious stimulus is a potentially visible stimulus (the energy and duration of which can be determined) that cannot be consciously perceived in a given test due to temporary distraction or inattention. Subliminal presentation is often achieved by masking, threshold stimuli, binocular rivalry, and continuous flash suppression, while the preconscious presentation is achieved by inattentional blindness and attentional blink (AB) (Dehaene and Changeux, 2011).

Among the above paradigms, the AB facilitates the study of the mechanisms that affect the rate and depth of information processing in various setups and therefore provides an elegant way to study correlations of conscious perception (Janson and Kranczioch, 2011). It also offers an optimal way to contrast the neural fate of identical stimuli; they are consciously perceived in only some of the trials and fail in other trials (Sergent et al., 2005). The AB effect is defined as the reduced ability to report a second target (T2) after identifying the first target (T1) in a rapid serial visual presentation (RSVP) of stimuli (Raymond et al., 1992). This “blink” occurs when T2 appears in the time window of 200–500 ms after the T1 presentation. This phenomenon has spawned quite a several theories on the origins of the “blink” during the past two decades.

Early models of the origins of the AB were focused more on the central capacity limitations, such as the two-stage model (Chun and Potter, 1995; Jolicoeur, 1998, 1999; Bowman and Wyble, 2007), the interference in retrieving theory (Duncan et al., 1994; Shapiro et al., 1994), and suspending processing theory (Raymond et al., 1992). More recently, theories have shifted from assuming central capacity limitations in memory consolidation toward an emphasis on the configuration of the attention network, such as delayed attentional reengagement accounts (Nieuwenstein et al., 2005; Nieuwenstein, 2006). This theory proposes that the AB is the result of the dynamics of attentional selection—a top-down process that dictates that attention is engaged to T1 and disengaged as soon as T1 disappears cannot react fast enough for the re-engagement to T2. Similar theories, such as the overinvestment theory (Olivers and Nieuwenhuis, 2006; Shapiro et al., 2006) and the global workspace model for AB (Dehaene et al., 2003) are also models emphasizing attentional control.

However, the question of why an AB is obtained in some trials but not in others has not yet been resolved. What is the main cause of this difference? Is it more attributed to the top-down attentional control process before the emergence of T2 and even before T1, or to the occupation of central capacity after the emergence of T1?

To test this assumption, studies have explored the neural cognitive mechanism preceding T2 utilizing various techniques. For instance, studies drawing on electroencephalographic (EEG) or magnetoencephalography (MEG) recording techniques set to measure the brain oscillation mechanism of AB have the advantage of high resolution in the time domain. The majority of previous studies using the EEG recording technique explored the neural mechanism of AB in terms of the event-related potential (ERP) method, and they were most focused on the components induced by stimuli in the RSVP stream (Vogel et al., 1998; Vogel and Luck, 2002; Kranczioch et al., 2003; Sergent et al., 2005; Hommel et al., 2006). Under this framework, ERP evidence was more consistent with the early central capacity limitation assumption of AB. For instance, the T2-induced P3 component in the frontal or central cortical region was larger in T2-reported trials than it in T2-missed trials (Vogel et al., 1998), while T1-elicited P3 was smaller in T2-detected trials (Slagter et al., 2007). The divergence of T2-detected and missed conditions began to appear at approximately 270 ms (Sergent et al., 2005).

A problem exists that the ERP technique is locked to the stimuli and nearly all studies using ERP focused on the process after stimuli appeared. Additionally, the ERP components are more indicative of a certain brain area activated or inhibited during the cognitive process. Nonetheless, the working mode of our brain is more like a collaboration of distributed neural groups. Different neural assemblies communicate with each other in a given spatial and temporal structure, which is largely organized by oscillations (Draguhn and Buzsáki, 2004). Brain oscillations in different brain areas become synchronized and consequently allow for concerted execution of the whole range of brain functions (Arnal and Giraud, 2012; Chakravarthi and VanRullen, 2012). Thus, it is necessary to utilize more techniques in addition to ERP to explore the working mechanisms of our brain from a more comprehensive and holistic viewpoint. Fewer studies have focused on the brain synchronizations among different neural assemblies, especially for the time preceding target onset (Gross et al., 2004; Hipp et al., 2011).

To relate the temporal dynamics of attention network communication to observed attentional limitations in AB, a prime candidate for communication among distributed systems in our brain is neural synchronization (Singer, 1999). Phase-locking value (PLV), also known as a phase-locking index (PLI), is one of the most widely used parameters in quantifying neural synchronization (Lachaux et al., 1999). We introduced this concept in detail in the “Materials and Methods” section. A few studies have tried to predict the behavioral performance of T2 using PLV. For instance, Gross et al. (2004) reported that beta synchronization in the target-related network is significantly stronger during the entire RSVP stream in the T2-correct condition than in its incorrect condition; and beta synchronization is significantly stronger to targets and significantly weaker to distractors (Gross et al., 2004). Additionally, high levels of alpha PLV predicted a miss, whereas low levels predicted correct perception of the T2 (Kranczioch et al., 2007). It was assumed that oscillatory neuronal synchronization mediates neuronal communication within frequency-specific, large-scale cortical networks (Hipp et al., 2011).

Based on previous studies using PLV measurement, we moved forward to combine the PLV and new data mining strategies of machine-learning methods to predict target performance. Among the existing machine-learning methods, support vector machines (SVMs) have demonstrated superior performance in several domains and, in particular, in neuroscience (Liu et al., 2009; Cerasa et al., 2015). SVMs are designed to exhibit the desirable theoretical property and advantage of maximizing the margin between classes to provide good generalization and thus, can yield reliable results using a minimal amount of data for training (Krusienski et al., 2006). We utilized the SVM algorithm to differentiate the trials in which both T1 and T2 were correctly reported and those in which only T1 was reported but T2 was missed.

In summary, we aimed to explore whether the pre-stimulus neural states in our brain could predict the final target performance and how far it can be achieved. We hypothesized that the different attentional states leading to different behavioral outcomes (T2 reported correctly or not) are characterized by specific patterns of synchronization between distributed brain areas involved in target processing. Here, we tested the assumption by adopting the AB paradigm and analyzed interarea synchronization based on the PLV parameter. Specifically, brain states before T1 presentation and during T1–T2 should determine the detection of T2 in the AB interval. Furthermore, the resources allocated to T1 and T2 processing should be directly mirrored by the P3 component, which means that the successful temporal management of resources should be reflected in larger T2-induced P3 and smaller in T1-elicited P3 for detected T2 trials (Vogel et al., 1998; Slagter et al., 2007).

## Materials and Methods

### Subject

Eighteen male participants were recruited at the Zhejiang University, with an average age of 21.35 (20–22). They were required to be free of current or past neurological or psychiatric disorders. All participants had normal or corrected-to-normal visual acuity and normal color vision, and all participants were right-handed and Chinese native speakers. They were paid for their participation and informed written consent was obtained prior to the start of the experiment. Two participants were excluded from the EEG data analysis due to excessive head movements, thus 16 participants remained. The smallest sample size of subjects was calculated as 12 by using G-power 3.1.9.7, with predefined effect size set as 0.4, statistical test power set as 0.8 in a within-subject repeated ANOVA design.

### Stimuli and Procedure

According to previous research, there are three preconditions for AB to be observed: (i) the stimulus presentation rate must be approximately 10 items per second. (ii) At least one distracter must follow T1 and T2. (iii) The second target, T2, must be presented between approximately 100 and 500 ms after T1 (Raymond et al., 1992; Klimesch, 2012). Therefore, our experiment was designed as follows: visual stimuli were presented on a CRT computer monitor with a refresh rate of 80 Hz, resulting in a frame duration of 12.5 ms. Each trial consisted of an RSVP stream of letters and digits. Distractors were letters and the two targets were digits. Both T1 and T2 appeared in every single trial. Visual stimuli were subtending 0.36 by 0.50 of visual angle at a viewing distance of approximately 80 cm and were presented as fixed on a white background.

**FIGURE 1:**
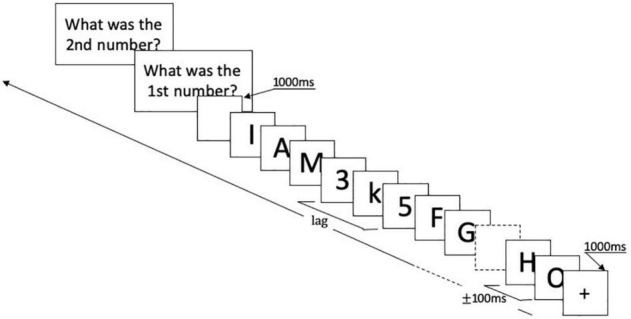
Experimental design. Each trial started with a black fixation cross lasting for 1,000 ms. Then, the RSVP sequence began: T1 was presented after 8–15 distractors, followed by 10 more items in which T2 appeared among the distractors. T2 could be the first, third, or seventh item following T1. Items in RSVP were presented for 50 ms each, and stimulus-onset-asynchrony (SOA) was 100 ms. Following the RSVP sequence, the screen remained blank for 1,000 ms. Then, responses were requested for T1 and T2 *via* response screens. RSVP, rapid serial visual presentation.

Stimuli were presented at the center of a gray screen. At the start of each trial, a black fixation cross appeared for 1,000 ms. Then, the RSVP sequence began: T1 was presented after 8–15 distractors, followed by 10 more items in which T2 appeared among the distractors. T2 could be the first, third, or the seventh item following T1, corresponding to the conditions of Lag1, Lag3, and Lag7. Items in RSVP were presented for 50 ms each, and stimulus-onset-asynchrony (SOA) was 100 ms. Following the RSVP sequence, the screen remained blank for 1,000 ms. Subsequently, participants were required to report T1 and T2 by clicking the corresponding key on the keyboard. There were 80 trials for each lag condition, and the total number of experimental trials was 240. All the lag conditions were randomized and presented in four blocks. Short breaks were scheduled between four blocks. Stimuli and trial structure are illustrated in Figure 1.

### Electroencephalographic Recording

Participants were tested individually in a soundproof and electrically shielded recording booth. They were asked to fixate their gaze on the center of the monitor as much as possible, to minimize movement of eyes and facial muscles, and to keep their bodies (especially their heads) as still as possible during the presentation. Continuous EEG was recorded using the Neuroscan SynAmps2 system at a sampling rate of 1,000 Hz and analog-filtered DC-100 Hz. Quick-Cap 64 electrode (Ag/AgCl) sites were placed according to an international modified 10–20 system montage (Nuwer et al., 1999). Additionally, four bipolar electrooculogram (EOG) signals to monitor eye movements were set. Impedances were kept below 10 kW for all electrodes. All scalp electrodes and the EOG signals were referenced to the left mastoid (M1) during recording. All scalp electrodes were grounded at a point midway between Fpz and Fz.

### Preprocessing Analysis for Electroencephalographic Data

Off-line analysis was performed using MATLAB. Ocular artifacts (such as horizontal and vertical EOG) were regressed out using the least-squares method (Jin et al., 2018). In addition, we rejected artifacts with a standard of ±80 μV and artifacts with a gap between the maximum and minimum value exceeding 120 μV. After artifact rejection, an average of over 72 trials was left for each condition per subject, with conditions not being significantly different from each other (Keil et al., 2014). EEG data were filtered in a band of (0.5, 40). Epochs were extracted from continuously recorded EEG relative to the onset of T1s, 800 ms before and 1,000 ms after the stimuli. The mean voltage of 800 ms segments preceding stimuli was subtracted as the baseline. The averaged ERP waveforms were time-locked to the onset of T1. Trials with incorrect T1 responses were excluded from the ERP waveforms and all behavioral analyses. We only analyzed the Lag3 data from T1 correct epochs and then classified them into the correct T2 and incorrect T2 epochs according to the behavioral results.

### Event-Related Potential Analysis for P3 Component

We analyzed the average amplitude from T1 onset (referenced as time zero) in a time window of 100 ms, which progresses at a steady pace of every 10 ms. The difference between T2-detected and T2-undetected conditions was calculated using a *t*-test for each window. The significant results of *t*-tests are marked with shadows in the waveforms of each electrode. The onset of the P3 was measured using the fractional area latency of the component, which was defined as the time point at which the waveform reached 25% of its area within the time window of 300–900 ms post-stimulus (Hansen and Hillyard, 1984). Time windows were defined based on previous studies (Kranczioch et al., 2003, 2007) and visual inspection to cover the main properties of the P3 processes.

### Phase Coupling Analysis

Phase indicates a particular time point within a single oscillatory cycle or period and is frequently measured in degrees (0–2π). Phase coupling or synchronization assesses the stability of differences between phases of EEG signals at equivalent frequencies taken simultaneously by different electrodes. More simply stated, it is a measure of how the relative phase is distributed over the unit circle (Bosch et al., 2018). Phase coupling has been interpreted as a means of communication between distant neural assemblies (Lachaux et al., 1999; Varela et al., 2001; Fries, 2005; Uhlhaas et al., 2009). This assumption has been lent more credibility with evidence from intracranial studies in primates (Canolty et al., 2010). Previous AB research using PLV (Kranczioch et al., 2005), phase synchrony index (SI; Gross et al., 2004), and dynamic cross-lag phase synchronization (dI; Nakatani et al., 2005) all indicated the neural synchronization of various brain areas and will be referred to as interarea phase synchronization.

To discover to what extent two sensor locations were synchronized, we used the PLV, which is one of the most widely used measures of brain synchronization (Lachaux et al., 1999; Varela et al., 2001). It quantifies the phase relationship between two signals with high temporal resolution without making any statistical assumptions on the data.

Given two time series of signals *x*(*t*) and *y*(*t*) and a frequency of interest *f*, the procedure computes a measure of phase locking between the electrodes of *x*(*t*) and *y*(*t*) for each time point at frequency *f*. This process requires the extraction of the instantaneous phase of every signal at the target frequency. The phases are calculated by convolving each signal with a complex wavelet function (pn_eegPLV, written by: Praneeth Namburi) (Lachaux et al., 1999).

Under certain frequencies (alpha or beta), PLV serials (1,891 × 1,800 × 2) in two-time windows were chosen for analysis: (−300, 0) and (0, 300) with the T1 onset as a reference, separately for T2-detected and undetected conditions. The parameter 1,891 stands for the number of electrode pairs, 1,800 stands for the time span (we chose the epoch 800 ms before and 1,000 ms after T1 onset), and 2 stands for the two conditions of T2 detected or not. The PLV values in the window were summed, and then the results of certain electrode pairs were kept as PLV [sum (*x*, *y*), *f*]. *x* and *y* indicate the corresponding electrodes, and *f* indicates the frequency. We calculated the PLVs in the alpha and beta frequency ranges in all the electrode pairs and then normalized them to minimize the influence of trial number imbalance under the two conditions. After this normalization, the key feature of PLV was sensitive only to phases, irrespective of the amplitude of each signal. Then *t*-tests (*p* < 0.005) between PLV (sum, *x*) in the T2-correct and incorrect trials were adopted to obtain the significant PLV pairs as features to perform the SVM test.

### Support Vector Machines Calculation

The SVM is designed to determine the hyperplane that maximizes the separating margin of separation between the two classes of a binary classification. With class labels coded as *yi* ∈ [±1], Eq. 1 can be reformulated as


(1) $$y_{i}\,\left(w\cdot f\,\left(x_{i}\right)+b\right)+\eta_{i}\geq 1$$

Where η*_*i*_* > 0 represents the distance from the misclassified points to the margin. With the margin simply equaling 2/*w*, the maximum margin will minimize subject to Eq. 2:


(2) $$C\,\sum_{i=1}^{l}\eta_{i}+0.5\,{∥w∥}^{2}$$

Subject to Eq. 2, where *C* is an arbitrary regularization parameter that reflects the penalty for misclassification and *l* is the number of training examples. This constrained optimization problem can be solved using Lagrangian multipliers, equivalently maximizing:


$$\begin{array}{c} \text{max}\,\left(\alpha \right)\,\left\{\sum_{i=1}^{l}\alpha_{i}-\frac{1}{2}\,\sum_{i=1}^{l}\sum_{j=1}^{l}\alpha_{i}\,\alpha_{j}\,y_{i}\,y_{j}\,K\,\left(x_{j},x_{i}\right)\right\}\\ s.t.\alpha_{i}\geq 0,i=1,\ldots ,l\\ \sum_{i=1}^{l}\alpha_{i}\,y_{i}=0 \end{array}$$

Where the kernel function *K*(*xj*, *xi*) = Ø (*xj*) and Ø (*xi*) defines the non-linear transformation and Ø (*x*) = *x* for the linear case. With the vector of Lagrangian multipliers, the classification score of a feature vector *x*, disregarding the inconsequential bias term, is computed as follows:


$${\text{score}}_{\text{SVM}}=\sum_{i=1}^{l}\alpha_{i}\,y_{i}\,K\,\left(x_{i},x\right)$$

The reason we did not choose a non-linear SVM, such as Gaussian SVM, is that overfitting can be a common dilemma with non-linear classifiers. Although they are often able to model the training data very accurately, they can fail if the training data are not wholly representative of independent test data. Overfitting may be resolved by tuning the classification algorithm to generalize to independent test data, leading to another drawback of SVMs—the onerous process of attaining a suitable model and training parameters. Because SVM parameters, such as the regularization parameter and kernel bandwidth, cannot be intuitively generated, it may be necessary to examine the multiple combinations to achieve optimal performance.

Importantly, in all cases, the data were divided into two non-overlapping sets, a training set, and a test set. The Leave One Out Cross-Validation (LOOCV) approach was used due to its advantages of producing model estimates with less bias and more ease in smaller samples. LOOCV consists of splitting the dataset randomly into *n* partitions. At each of the *n*-th iterations, *n* − 1 partitions will be used as the training set, and the remaining sample will be used as the test set. At each of the *n*-th iterations, the whole data set is used as the training set except for one sample, which is left out as a test set.

The performance of the classifier was evaluated by comparing the classifier predictions relative to the test data with the actual behavioral results obtained from the test data. In the case of binary classification, the chance level is 50% and the maximum classification performance is 100%. We also used the area under the ROC (AUC) to measuring classifier performance based on sensitivity and specificity (Kassraian-Fard et al., 2016).

## Results

### Behavioral Results

**FIGURE 2:**
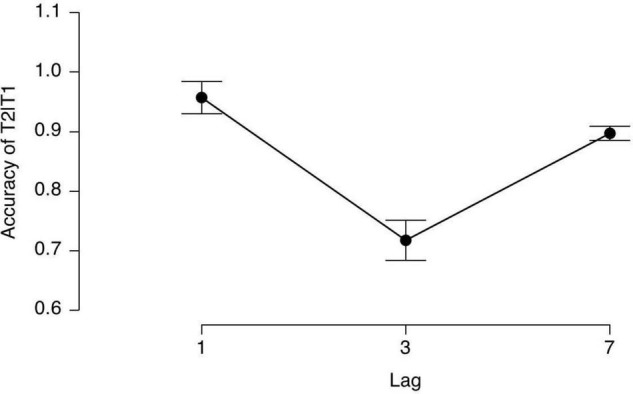
The averaged T2 accuracy in every lag condition given T1 was correct.

According to previous studies, we calculated the T2 accuracies under three lag conditions premised on a correct T1 response. The average accuracies (mean) and SD in every lag condition are shown in Figure 2.

A repeated ANOVA indicated a significant main effect of Lag [*F*(2, 15) = 23.35, *p* < 0.0001, η^2^ = 0.61], indicating that the AB did occur in our study. The *post hoc* comparisons showed that the accuracies in Lag1 and Lag7 were significantly higher than that in Lag3 (*p* < 0.001, *p* < 0.001), but there was no difference between Lag 1 and Lag7. All the *p*-values were corrected by a Bonferroni correction.

### Event-Related Potential Results

**FIGURE 3:**
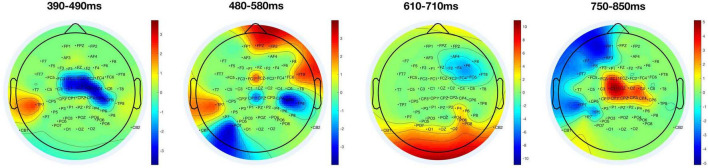
Distribution of significant areas depending on T2 detection. Significant results of *t*-tests (*p* < 0.05) between T2-detected and T2-undetected conditions are displayed on 2D brain maps in four-time windows. The red parts show that the average amplitudes in the T2-detected condition are larger than in the T2-undetected condition; and the blue parts represent the opposite situation.

According to our observations and previous related research (McArthur et al., 1999), the T1-related P3 had a peak latency of approximately 400 ms, while the T2-related P3 had a peak latency of approximately 600 ms. We calculated the averaged amplitudes of a 100 ms time window separately for T2-detected and T2-undetected conditions and moved forward at a pace of 10 ms. The distribution of significant results for *t*-test (*p* < 0.05) conditions was displayed on a 2D brain map in four-time windows as shown in Figure 3.

As we can see from Figure 3, the red parts indicate that the average amplitudes in the T2-detected condition are larger than those in the T2-undetected condition and vice versa. Thus, the central part of the brain (mainly covered C1, Cz, C2, FCz, and FC1) is sensitive to T2 detection. In detail, in the 390–490-ms time window, the central part is blue, and in contrast, it turned red in the 750–850-ms time window. The former time window may correspond to the T1-related P3 peak, and the latter may correspond to the T2-related P3 peak.

**FIGURE 4:**
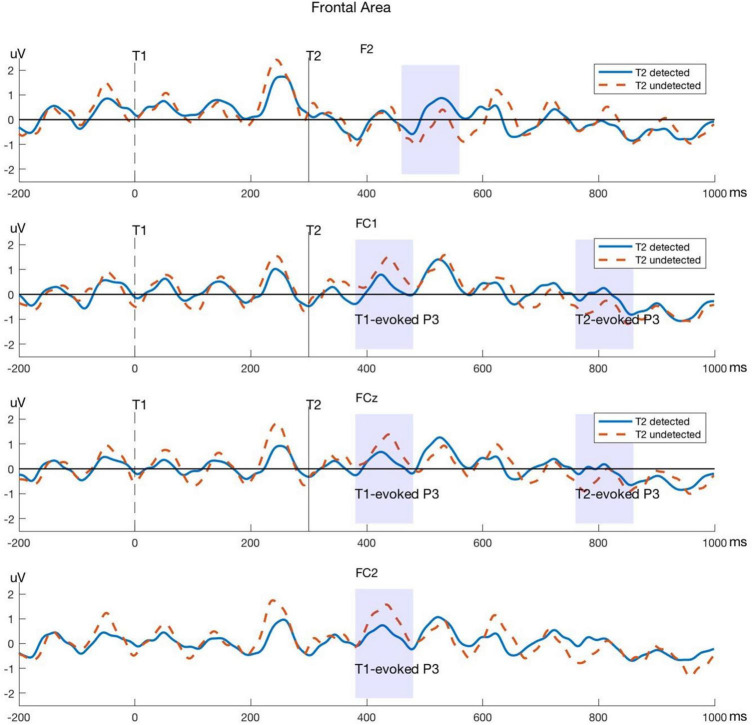
Typical P3 components evoked by T1 and T2 in electrodes within the frontal area. The blue lines represent the T2-detected condition and the red dashed lines represent the T2-undetected condition. Significant differences in average amplitude between the two conditions are shown in shadows as above.

**FIGURE 5:**
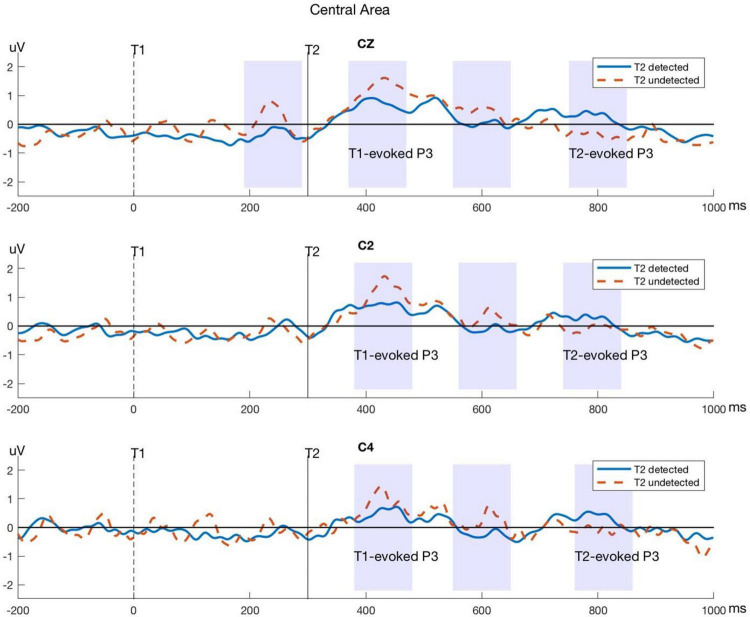
Typical P3 components evoked by T1 and T2 in electrodes within the central area. The blue lines represent the T2-detected condition and the red dashed lines represent the T2-undetected condition. Significant differences in average amplitude between the two conditions are shown in shadows as above.

Significant differences between T2-detected and T2-undetected conditions in the average amplitudes of P3 time windows in the frontal and central regions are marked with shadows in the corresponding ERP waveforms and are shown in Figures 4, 5.

Moreover, we used a permutation method to test the significance of the difference between detected and undetected conditions for the T1- and T2-evoked P3 components in the corresponding electrodes in the frontal and central areas. The detailed *p*-values of P3 components in every electrode are shown in Table 1.

**TABLE 1:** Corresponding permutation *p*-values of T1- and T2-evoked P3 components in average amplitudes difference between T2-detected and -undetected conditions.

	**FC1**	**FC2**	**FCz**	**CZ**	**C2**	**C4**
T1 evoked P3 (380–480 ms)	0.012	0.014	0.018	0.0036	0.0039	0.0039
T2 evoked P3 (760–960 ms)	0.033	0.11	0.023	0.0001	0.007	0.008

### Phase-Locking Value and Corresponding Support Vector Machines Results

**FIGURE 6:**
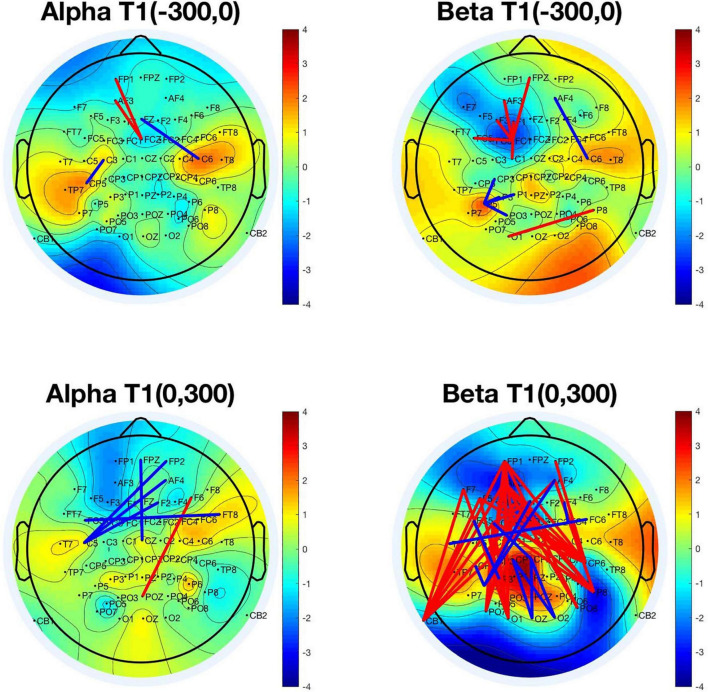
Significant phase-locking value (PLV) pairs were used in differentiating T2-correct trials and T2-incorrect trials. The *upper left panel* represents the significant PLV pairs in the time window (–300, 0) with T1 onset as time 0 in the alpha frequency band. The *upper right panel* represents the time window of (–300, 0) in the beta frequency band. The *bottom left panel* represents the significant PLV pairs in the time window of (0, 300) in the alpha frequency band. The *bottom right panel* represents the significant PLV pairs in the time window of (0, 300) in the beta frequency band (red lines show higher PLV values in T2-correct trials, and blue lines reflect the opposite situation). The colors in the topographical map of the brain were obtained by calculating the PLV means of every electrode with all the other electrodes. PLV, phase-locking value.

We chose PLV serials (1,891 × 1,800 × 2) in two-time windows for analysis: (−300, 0) and (0, 300) with the T1 onset as a reference, separately for T2-detected and -undetected conditions under a certain frequency (alpha or beta). Then *t*-tests (*p* < 0.005) for PLVs in the T2-correct and -incorrect trials were adopted to obtain the significant values after normalization. All the significant pairs are depicted in the 2D brain map shown in Figure 6 (red lines show higher PLV values in T2-correct trials, and blue lines reflect the opposite situation).

Under the alpha frequency band in the time window of (−300, 0), four pairs of electrodes were found to be significantly different in their PLV by using a *t*-test to compare T2-detected and -undetected trials. These pairs were FP1-FCz, AF3-FCz, Fz-C6, and C3-CP5.

Under the beta frequency band in the time window of (−300, 0), 11 pairs of electrodes were found to be significantly different in their PLV by using the same method. These pairs were FPz-FC1, AF3-FC1, F3-FC1, F1-FC1, FC5-FC1, C1-FC1, AF4-C6, P5-CP3, P5-P1, P5-PO3, and P8-O1.

Under the alpha frequency band in the time window of (0, 300), six pairs of electrodes were found to be significantly different in their PLV. These pairs were (FPz, FP2, AF4, F2, FC5, FT8, C5, and Cz), with a decrease in alpha synchronization in the T2-detected condition. These connections were between the right prefrontal and left central areas, and bilateral frontal connections. Only one connection (F6-POz) was increased before T1 onset in the T2-detected condition.

Under the beta frequency band in the time window of (0, 300), 47 pairs of electrodes were found significantly different in their PLVs. Figure 5 shows that these pairs covered a broad area of the brain, such as the frontal, temporal, parietal, and occipital regions. The majority of PLV pairs were long-range connections from the frontal to the posterior part of the brain, with increased synchronization in the T2-correct condition. The minority of PLV pairs were more local in the parietal and central areas, with a decreased synchronization in the T2-correct condition.

Then, we used different combinations of these pairs as predicting factors in the SVM function separately for the four conditions. The SVM calculation results demonstrated that these factors can predict the performance of the T2 target with high accuracy. Table 2 indicates the results of different combinations of features in the SVM algorithm.

**TABLE 2:** Results of different combinations of PLV pairs in SVM calculation.

	**PLV pairs as features**	**Max accuracy**	**Max AUC**
Alpha T1 (-300, 0)	(FP1,FCz), (AF3, FCz), (Fz, C6), and (C3, CP5)	0.8125	0.8633
Beta T1 (-300, 0)	(FPz, FC1), (FC5, FC1), (CP3, P5), and (P5,P1)	0.8750	0.8984
Alpha T1 (0, 300)	(FC5, FT8), (F2, C5), (FPz, Cz), and (F6, POz)	0.8750	0.9063
Beta T1 (0, 300)	(FC6, T7), (F1, CP6), (FC3, CP6), and (FC3, CPz)	0.9688	1

*PLV, phase-locking value; SVM, support vector machine.*

### Regression Between P3 and Preceding Phase-Locking Value

To explore whether the P3 amplitude could be predicted by preceding PLV activities, we also carried out regressions between P3 average amplitudes and preceding PLVs separately in different frequency bands. In detail, we analyzed the regression between PLV in the time window of (−300, 0) and T1-induced P3 and the regression between PLV in the time window of (0, 300) and T2-induced P3 in the alpha and beta frequency bands. We did not go through all the electrodes; instead, we chose electrodes based on their significance in differentiating T2-detected and T2-undetected conditions.

Significant regression results in the time window (−300, 0) and corresponding electrodes are shown in Table 3.

**TABLE 3:** Results of significant regressions between PLV pairs and P3 in the time window (−300, 0).

	**PLV (**-**300, 0)**	**T1-induced P3**	**R2**	**P**
Alpha	FP1, FCz	FCz	0.175	0.017
	AF3, FCz	FCz	0.151	0.028
Beta	F1, C1	FC2	0.174	0.017
	AF4, C6	P2	0.143	0.033

*PLV, phase-locking value.*

Significant regression results in the time window (0, 300) and corresponding electrodes are shown in Table 4.

**TABLE 4:** Results of significant regressions between PLV pairs and P3 in the time window (0, 300).

	**PLV (0, 300)**	**T2-induced P3**	**R2**	**P**
Alpha	FP2,C5	Cz	0.13	0.042
	AF4, C5	C2	0.125	0.047
	AF4, C5	C4	0.156	0.025
	F2, C5	Cz	0.144	0.032
	F2, C5	C2	0.204	0.009
	F2, C5	C4	0.153	0.027
	F6, POz	Cz	0.175	0.017
Beta	Fz, FC3	C4	0.149	0.029
	FP1, FC1	C2	0.181	0.015
	FP1, FC1	C4	0.207	0.008
	AF4, FC1	C2	0.146	0.031
	AF4, FC1	C4	0.200	0.01
	F5,CP6	C4	0.170	0.019
	F1,CP6	C4	0.144	0.032
	FC3, CP6	C4	0.172	0.018
	CP5,P5	C4	0.136	0.037
	FC5, P3	FC1	0.132	0.04
	FC5, P3	FCz	0.128	0.044
	AF3, P8	Cz	0.125	0.047
	AF4, P8	Cz	0.0266	0.0025
	FC3, P8	FCz	0.124	0.049
	FCz, P8	FCz	0.170	0.019
	FCz, P8	Cz	0.130	0.043
	FP1, CB1	Cz	0.142	0.034
	AF3, CB1	Cz	0.136	0.038
	F5, CB1	Cz	0.131	0.041
	FC3, CB1	FCz	0.131	0.041
	FCz, CB1	FCz	0.144	0.032
	FCz, CB1	Cz	0.163	0.022
	FP1, O1	Cz	0.161	0.023
	FP1, O1	C2	0.163	0.022
	AF3, O1	Cz	0.162	0.022
	AF3, O1	C2	0.154	0.026
	AF3, O1	C4	0.126	0.046
	CPz, O2	C4	0.156	0.025

*PLV, phase-locking value.*

## Discussion

The main aim of this study was to explore whether the pre-stimulus neural states in our brain could predict the final target performance utilizing the AB paradigm. The mechanisms of our brain controlling why physically identical information sometimes succeeds in reaching awareness while failing at other times remain poorly understood. We calculated the phase synchronization to predict behavioral outcomes using an SVM classifier and examined the correspondence between ERP components and behavioral performance. The present results showed that the pre-T2 PLV could predict the later T2 performance with high accuracy and support the attention control hypothesis that certain attentional states and low efficiency in attentional resource allocation lead to the “blink”.

### Phase Coupling Results With the Support Vector Machines Learning Algorithm

Recently, there has been an increase in the number of studies applying data mining tools to neuroscience (Squarcina et al., 2017). However, the vast majority of these studies commonly seek to discover patterns in electrophysiological signals and images correlated with the diagnosis, prognosis, and progress of a particular pathology or brain disorder and with the image analysis of normal/disease resting-state functional magnetic resonance imaging (fMRI) (Rashid et al., 2016; Hojjati et al., 2017). By comparison, very few investigations in this area use machine-learning techniques for studying normal brain cognitive functions. In particular, tools and approaches tailored to grasp the complexity of brain electric activity through the analysis of EEG signals are lacking (Bosch et al., 2018).

Our primary finding demonstrates that classification of EEG PLV patterns using data mining tools with neural cognitive data related to consciousness is achievable. For each pair of EEG channels, phase synchrony was calculated within a given time window, separately for detected and undetected T2 trials. A novel feature selection methodology that identifies the most relevant connections in these networks was applied with a linear SVM-based classifier. This methodology allows us to determine the most relevant connections, thus reducing the complexity, and facilitating the interpretation of mined patterns.

According to our result, the most significant connections were observed in electrode pairs covering the parietal, temporal, and frontal areas. According to previous research, these areas are linked to attentional control in general (Marois and Chun, 2000; Dehaene et al., 2003; Friedman-Hill et al., 2003).

Interestingly, interarea phase coupling varied among different frequency bands and different time windows. In the alpha band, we observed long-range connections, while in the beta band, the phase coupling only showed small-area synchronization, thus supporting the theory of frequency-related brain function. This theory assumes that different brain networks oscillate at different frequencies, with small networks oscillating at fast frequencies (>40 Hz) and large networks oscillating at slower frequencies (<20 Hz) (Steinu and Von Sarnthein, 2000; Draguhn and Buzsáki, 2004). Slower oscillations may represent brain networks of a higher hierarchy, encompassing multiple lower-level networks, and thereby gating faster oscillations in a top-down manner (Lakatos et al., 2005, 2008). We discussed the phase coupling of alpha and beta frequencies in different time windows as follows.

#### Pre-T1 Time Window

Both the alpha and beta frequency activities could predict the behavioral outcomes in the pre-T1 time window. The alpha synchronization before T1 appearing in an RSVP stream could account for over 80% of the behavioral outcomes. In total, we found four pairs of significant neural connections: FP1-FCz, AF3-FCz, Fz-C6, and C3-CP5. The first two connections showed increased activity while the latter two showed decreased activity in the detected T2 condition.

Pre-T1 beta frequency activities could also function as a predictor of behavioral performance, with even higher accuracy. The significant pairs were FPz-FC1, AF3-FC1, F3-FC1, F1-FC1, FC5-FC1, C1-FC1, AF4-C6, P5-CP3, P5-P1, P5-PO3, and P8-O1. We saw that two electrodes (FC1 and P5) played a central role in interarea synchronization. The FC1-centered connections were increased while the P5-centered connections were decreased in the T2-correct condition. Our results were partly in line with those of Gross et al. (2004)’s research, where beta synchronization in the target-related network was significantly stronger during the entire stream in the non-AB condition than in the AB condition; and beta synchronization was significantly stronger for targets and significantly weaker for masks (Gross et al., 2004). Furthermore, the brain oscillation differences between detected and undetected T2, both beta frequency (15 Hz) in long-range neural synchrony and gamma frequency (40 Hz) ranges have been found to emerge even before target stimulus presentation (Gross et al., 2004; Nakatani et al., 2005). Using event-related fMRI techniques, Kranczioch et al. (2005) observed an increase in activation for the detected T2 condition during the lag between T1 and T2 in the frontal and parietal cortices. In contrast, in the occipitotemporal regions, activation was increased for missed T2 conditions (Kranczioch et al., 2005), which was consistent with our findings in beta frequency. They proposed that activation in the occipitotemporal regions might mainly reflect the duration of the attentive search, and the frontoparietal areas seem to be involved in a highly distributed network controlling visual awareness.

#### T1–T2 Interval Time Window

Phase coupling in the alpha band between T1 and T2 could predict 90% of the behavioral fate of T2 according to the SVM calculation. These significant neural connections mainly covered the frontal and central regions of the brain (FPz, FP2, AF4, F2, FC5, FT8, C5, and Cz), with a decrease in alpha synchronization in the detected T2 condition. As shown in Figure 6, these connections were between the right prefrontal and left central areas, and bilateral frontal connections. However, one connection (F6—POz) in the alpha band was increased before T1 onset in the T2-detected condition.

Phase coupling in beta frequency during the T1–T2 time window covered a broad area of the brain, such as the frontal, temporal, parietal, and occipital regions. The majority of PLV pairs were long-range connections from the frontal to the posterior part of the brain, with increased synchronization in the T2-correct condition. The minority of PLV pairs were more local in the parietal and central areas, with a decreased synchronization in the T2-correct condition. Very importantly, in this frequency and time window, the SVM classifier found the highest accuracies and AUC, which was nearly 1.

The electrode pair-related areas in our results have been linked to visual attention (Nobre et al., 1997; Hopfinger et al., 2000; Marois and Chun, 2000; Corbetta and Shulman, 2002) and working memory (McCarthy et al., 1996; Quintana and Fuster, 1999; Fletcher and Henson, 2001). According to Desimone and Duncan (1995), such a network may exert attentional control by biasing the processing of incoming visual information: neural representations of target- or goal-related stimuli receive top-down (i.e., frontal) support, which increases their chances of winning the competition for selection and, hence, their impact on overt behavior. Specifically, the components of the attention network likely represent the neural components responsible for coding the task-related stimuli, for maintaining a template of the target, and for matching the former against the latter (Gross et al., 2004). In contrast, insufficient top-down control could lead to deficits in both the inhibition of distractors and the attenuation of T1 processing or to a slower succession of stable states and a lack of facilitation of T2 processing (Janson and Kranczioch, 2011).

Functional magnetic resonance imaging findings also generally support the distribution of brain area function in attention control. For instance, Marcantoni et al. (2003) observed increased activation in the inferotemporal, lateral frontal, left posterior parietal, and occipital cortex for T2 stimuli presented during the AB window (Marcantoni et al., 2003). The fMRI evidence thus far implies the involvement of a frontal-temporal-parietal network in the AB. These higher cortical areas likely modulate the activity in lower visual areas via iterative feedback loops (Janson and Kranczioch, 2011).

### Event-Related Potential Components Relating to Behavioral Results

Our results also provide strong evidence in the time domain, particularly, for the P3 component. The average amplitude was smaller for T1-evoked P3 and yet larger for T2-evoked P3 in T2-detected trials. This phenomenon could be observed in the frontal and parietal regions. In line with our findings, the amplitude of the P3 component evoked by T1 was found to be correlated with the size of the AB (Shapiro et al., 2006). The P3 evoked by T2, on the other hand, was shown to be reduced for undetected stimuli compared with detected stimuli (Kranczioch et al., 2003). It has been suggested that this differentiation is due to a competition between neural processes devoted to the processing of the two targets reflected in the P3 components (Sergent et al., 2005). Since detected task-relevant stimuli nearly always generate a P3 response, a prediction that can be drawn from these studies is that the competition for attention resources should be directly reflected in the T1- and T2-induced P3 amplitudes.

According to the distribution of T1-evoked P3, we observed a slightly right-lateralized frontal-parietal network, and in T2-evoked P3, this network shifted to a more posterior region. This distribution is consistent with a previous fMRI study of AB. For instance, Kranczioch et al. (2005) found that clusters in the right and especially the left inferior parietal lobule (IPL), especially in the left inferior frontal gyrus (IFG), and the left superior frontal gyrus/anterior cingulate cortex (SFG/ACC) were related to T2 detection (Kranczioch et al., 2005).

Taken together, the image that emerges from the P3 results suggests that whether the AB occurs is a result of attentional control or allocation between T1 and T2. This pattern of the control process began in the frontal area first and then shifted into the central and parietal regions.

### Event-Related Potential Components Regression to Phase-Locking Value Pairs

Under the assumption that the P3 component induced by T1 and T2 could represent the attention resources allocated to T1 and T2, it is also reasonable to infer that P3 amplitudes are influenced by preceding PLV activities. Thus, we performed linear regressions between P3 amplitudes and preceding PLV pairs. In detail, we analyzed the regression between PLV in the time window of (−300, 0) and T1-induced P3, and the regression between PLV in the time window of (0, 300) and T2-induced P3 in the alpha and beta frequency bands. The results indicated that P3 amplitudes in electrodes FCz, Cz, C2, and C4 could be significantly predicted by preceding PLV activities. These functional PLV pairs were mainly covered the frontal and central areas in the time window (−300, 0). In time window (0, 300), these PLV pairs extended to long-range connections that covered the frontal and posterior regions in the beta band.

Therefore, our attempt to explore the inner relationship between PLV activity, P3 amplitudes, and behavior has proven to be effective. The current results obtained from the three parts could be combined into a whole system in which the former could predict the latter. That is, whether a target (T2) could be detected would already be determined before it appears because of the preparation state of our brain, thus supporting the attention control theory. Top-down attention control accounts offer an important perspective to scrutinize the mechanism of our brain for processing stimuli under attention-limited conditions, which may also be applied to other fields of cognitive processes.

It should be acknowledged that there were limitations in our study. First, we only had male participants in our study, although there was little research reporting gender difference in the classic AB task, this gender distribution will limit the generalizability of the findings. Second, the number of our participants was not much higher than the minimum standard of sample size. Therefore, further research is needed to confirm and consolidate the current results.

## Conclusion

In summary, our results indicate that the phase synchronization in alpha and beta frequency bands preceding T2 could predict the behavioral performance in T2 detection. Additionally, the P3 components evoked by T1 and T2 could reflect the behavioral outcomes and showed high consistency with previous studies. These results supported that the AB is not the result of a processing impairment in a single particular process or brain area, but the consequence of a dynamic interplay between several processes and/or parts of a neural network. Our findings suggest an intriguing influence of the top-down attention control on simple cognitive processes and provide a new effective way of exploring the neural mechanism for this influence.

## Data Availability Statement

The raw data supporting the conclusion of this article will be made available by the authors, without undue reservation.

## Ethics Statement

The studies involving human participants were reviewed and approved by the Ethics Committee in Zhejiang University. The patients/participants provided their written informed consent to participate in this study.

## Author Contributions

YY wrote the manuscript. YW was in charge of the data processing and methods writing. TX contributed to the data process. FC contributed to the logic and frame of writing and is the corresponding author. All authors contributed to the article and approved the submitted version.

## Conflict of Interest

The authors declare that the research was conducted in the absence of any commercial or financial relationships that could be construed as a potential conflict of interest.

## Publisher’s Note

All claims expressed in this article are solely those of the authors and do not necessarily represent those of their affiliated organizations, or those of the publisher, the editors and the reviewers. Any product that may be evaluated in this article, or claim that may be made by its manufacturer, is not guaranteed or endorsed by the publisher.

## References

[B1] Arnal L. H. Giraud A.-L. (2012). Cortical oscillations and sensory predictions. *Trends Cogn. Sci.* 16 390–398. 10.1016/j.tics.2012.05.003 22682813

[B2] Benwell C. S. Y. Coldea A. Harvey M. Thut G. (2021). Low pre-stimulus EEG alpha power amplifies visual awareness but not visual sensitivity. *Eur. J. Neurosci.* 10.1111/ejn.15166 33655566

[B3] Bosch P. Herrera M. López J. Maldonado S. (2018). Mining EEG with SVM for understanding cognitive underpinnings of math problem solving strategies. *Behav. Neurol.* 2018:4638903. 10.1155/2018/4638903 29670667 PMC5835340

[B4] Bowman H. Wyble B. (2007). The simultaneous type, serial token model of temporal attention and working memory. *Psychol. Rev.* 114 38–70. 10.1037/0033-295X.114.1.38 17227181

[B5] Canolty R. T. Ganguly K. Kennerley S. W. Cadieu C. F. Koepsell K. Wallis J. D. (2010). Oscillatory phase coupling coordinates anatomically dispersed functional cell assemblies. *Proc. Natl. Acad. Sci. U. S. A.* 107 17356–17361. 10.1073/pnas.1008306107 20855620 PMC2951408

[B6] Cerasa A. Castiglioni I. Salvatore C. Funaro A. Martino I. Alfano S. (2015). Biomarkers of eating disorders using support vector machine analysis of structural neuroimaging data: preliminary results. *Behav. Neurol.* 2015:924814. 10.1155/2015/924814 26648660 PMC4663371

[B7] Chakravarthi R. VanRullen R. (2012). Conscious updating is a rhythmic process. *Proc. Natl. Acad. Sci. USA* 109 10599–10604. 10.1073/pnas.1121622109 22689974 PMC3387058

[B8] Chun M. M. Potter M. C. (1995). A two-stage model for multiple target detection in rapid serial visual presentation. *J. Exp. Psychol. Hum. Percept. Perform.* 21 109–127. 10.1037/0096-1523.21.1.109 7707027

[B9] Corbetta M. Shulman G. L. (2002). Control of goal-directed and stimulus-driven attention in the brain. *Nat. Rev. Neurosci.* 3 201–215. 10.1038/nrn755 11994752

[B10] Dehaene S. Changeux J. P. (2011). Experimental and theoretical approaches to conscious processing. *Neuron* 70 200–227. 10.1016/j.neuron.2011.03.018 21521609

[B11] Dehaene S. Changeux J.-P. Naccache L. Sackur J. Sergent C. (2006). Conscious, preconscious, and subliminal processing: a testable taxonomy. *Trends Cogn. Sci.* 10 204–211. 10.1016/j.tics.2006.03.007 16603406

[B12] Dehaene S. Sergent C. Changeux J.-P. (2003). A neuronal network model linking subjective reports and objective physiological data during conscious perception. *Proc. Natl. Acad. Sci. U.S.A.* 100 8520–8525. 10.1073/pnas.1332574100 12829797 PMC166261

[B13] Del Cul A. Baillet S. Dehaene S. (2007). Brain dynamics underlying the nonlinear threshold for access to consciousness. *PLoS Biol.* 5 2408–2423. 10.1371/journal.pbio.0050260 17896866 PMC1988856

[B14] Desimone R. Duncan J. (1995). Neural mechanisms of selective visual attention. *Annu. Rev. Neurosci.* 18 193–222. 10.1146/annurev.ne.18.030195.001205 7605061

[B15] Draguhn A. Buzsáki G. (2004). Neuronal oscillations in cortical networks. *Science* 304 1926–1930. 10.1126/science.1099745 15218136

[B16] Duncan J. Ward R. Shapiro K. (1994). Direct measurement of attentional dwell time in human vision. *Nature* 369 313–315. 10.1038/369313a0 8183369

[B17] Fletcher P. C. Henson R. N. A. (2001). Frontal lobes and human memory: insights from functional neuroimaging. *Brain* 124 849–881. 10.1093/brain/124.5.849 11335690

[B18] Friedman-Hill S. R. Robertson L. C. Desimone R. Ungerleider L. G. (2003). Posterior parietal cortex and the filtering of distractors. *Proc. Natl. Acad. Sci. USA* 100 4263–4268. 10.1073/pnas.0730772100 12646699 PMC153081

[B19] Fries P. (2005). A mechanism for cognitive dynamics: neuronal communication through neuronal coherence. *Trends Cogn. Ences* 9 474–480. 10.1016/j.tics.2005.08.011 16150631

[B20] Gross J. Schmitz F. Schnitzler I. Kessler K. Shapiro K. Hommel B. (2004). Modulation of long-range neural synchrony reflects temporal limitations of visual attention in humans. *Proc. Natl. Acad. Sci. USA* 101 13050–13055. 10.1073/pnas.0404944101 15328408 PMC516515

[B21] Hansen J. C. Hillyard S. A. (1984). Effects of stimulation rate and attribute cuing on event-related potentials during selective auditory attention. *Psychophysiology* 21 394–405. 6463171 10.1111/j.1469-8986.1984.tb00216.x

[B22] Hanslmayr S. Aslan A. Staudigl T. Klimesch W. Herrmann C. S. Bäuml K.-H. (2007). Prestimulus oscillations predict visual perception performance between and within subjects. *NeuroImage* 37 1465–1473. 10.1016/j.neuroimage.2007.07.011 17706433

[B23] Hanslmayr S. Gross J. Klimesch W. Shapiro K. L. (2011). The role of α oscillations in temporal attention. *Brain Res. Rev.* 67 331–343. 10.1016/j.brainresrev.2011.04.002 21592583

[B24] Hipp J. F. Engel A. K. Siegel M. (2011). Oscillatory synchronization in large-scale cortical networks predicts perception. *Neuron* 69 387–396. 10.1016/j.neuron.2010.12.027 21262474

[B25] Hojjati S. H. Ebrahimzadeh A. Khazaee A. Babajani-Feremi A. (2017). Predicting conversion from MCI to AD using resting-state fMRI, graph theoretical approach and SVM. *J. Neurosci. Methods* 282 69–80. 10.1016/j.jneumeth.2017.03.006 28286064

[B26] Hommel B. Kessler K. Schmitz F. Gross J. Akyürek E. Shapiro K. (2006). How the brain blinks: towards a neurocognitive model of the attentional blink. *Psychol. Res.* 70 425–435. 10.1007/s00426-005-0009-3 16237554

[B27] Hopfinger J. B. Buonocore M. H. Mangun G. R. (2000). The neural mechanisms of top-down attentional control. *Nat. Neurosci.* 3 284–291. 10.1038/72999 10700262

[B28] Janson J. Kranczioch C. (2011). Good vibrations, bad vibrations: Oscillatory brain activity in the attentional blink. *Adv. Cogn. Psychol.* 7 92–107. 10.2478/v10053-008-0089-x 22253672 PMC3259030

[B29] Jin P. Zou J. Zhou T. Ding N. (2018). Eye activity tracks task-relevant structures during speech and auditory sequence perception. *Nat. Commun.* 9:5374. 10.1038/s41467-018-07773-y 30560906 PMC6299078

[B30] Jolicoeur P. (1998). Modulation of the attentional blink by on-line response selection: evidence from speeded and unspeeded Task1 decisions. *Memory Cogn.* 26 1014–1032. 10.3758/BF03201180 9796233

[B31] Jolicoeur P. (1999). Concurrent response-selection demands modulate the attentional blink. *J. Exp. Psychol. Hum. Percept. Perform.* 25:1097. 10.1037/0096-1523.25.4.1097 10681214

[B32] Kanai R. Walsh V. Tseng C. (2010). Subjective discriminability of invisibility: a framework for distinguishing perceptual and attentional failures of awareness. *Conscious Cogn.* 19 1045–1057. 10.1016/j.concog.2010.06.003 20598906

[B33] Kassraian-Fard P. Matthis C. Balsters J. H. Maathuis M. H. Wenderoth N. (2016). Promises, pitfalls, and basic guidelines for applying machine learning classifiers to psychiatric imaging data, with autism as an example. *Front. Psychiatry* 7:177. 10.3389/fpsyt.2016.00177 27990125 PMC5133050

[B34] Keil A. Debener S. Gratton G. Junghöfer M. Kappenman E. S. Luck S. J. (2014). Committee report: publication guidelines and recommendations for studies using electroencephalography and magnetoencephalography. *Psychophysiology* 51 1–21. 10.1111/psyp.12147 24147581

[B35] Kim C. Y. Blake R. (2005). Psychophysical magic: rendering the visible “invisible.”. *Trends Cogn. Sci.* 9 381–388. 10.1016/j.tics.2005.06.012 16006172

[B36] Klimesch W. (2012). Alpha-band oscillations, attention, and controlled access to stored information. *Trends Cogn. Sci.* 16 606–617. 10.1016/j.tics.2012.10.007 23141428 PMC3507158

[B37] Kranczioch C. Debener S. Engel A. K. (2003). Event-related potential correlates of the attentional blink phenomenon. brain research. *Cogn. Brain Res.* 17 177–187. 10.1016/S0926-6410(03)00092-2 12763203

[B38] Kranczioch C. Debener S. Maye A. Engel A. K. (2007). Temporal dynamics of access to consciousness in the attentional blink. *NeuroImage* 37 947–955. 10.1016/j.neuroimage.2007.05.044 17629501

[B39] Kranczioch C. Debener S. Schwarzbach J. Goebel R. Engel A. K. (2005). Neural correlates of conscious perception in the attentional blink. *Neuroimage* 24 704–714. 10.1016/j.neuroimage.2004.09.024 15652305

[B40] Krusienski D. J. Sellers E. W. Cabestaing F. Bayoudh S. McFarland D. J. Vaughan T. M. (2006). A comparison of classification techniques for the P300 Speller. *J. Neural Eng.* 3 299–305. 10.1088/1741-2560/3/4/007 17124334

[B41] Lachaux J. P. Rodriguez E. Martinerie J. Varela F. J. (1999). Measuring phase synchrony in brain signals. *Hum. Brain Mapp.* 8 194–208. 10.1002/(SICI)1097-0193(1999)8:4<194::AID-HBM4>3.0.CO;2-C 10619414 PMC6873296

[B42] Lakatos P. Karmos G. Mehta A. D. Ulbert I. Schroeder C. E. (2008). Entrainment of neuronal oscillations as a mechanism of attentional selection. *Science* 320 110–113. 10.1126/science.1154735 18388295

[B43] Lakatos P. Shah A. S. Knuth K. H. Ulbert I. Karmos G. Schroeder C. E. (2005). Lakatos, P. et al. an oscillatory hierarchy controlling neuronal excitability and stimulus processing in the auditory cortex. *J. Neurophysiol.* 94 1904–1911. 10.1152/jn.00263.2005 15901760

[B44] Liu H. Agam Y. Madsen J. R. Kreiman G. (2009). Timing, timing, timing: fast decoding of object information from intracranial field potentials in human visual cortex. *Neuron* 62 281–290. 10.1016/j.neuron.2009.02.025 19409272 PMC2921507

[B45] Marcantoni W. S. Lepage M. Beaudoin G. Bourgouin P. Richer F. (2003). Neural correlates of dual task interference in rapid visual streams: An fMRI study. *Brain Cogn.* 53 318–321. 10.1016/S0278-2626(03)00134-9 14607172

[B46] Marois R. Chun M. M. (2000). Neural correlates of the attentional blink. *Neuron* 28 299–308. 10.1016/S0896-6273(00)00104-5 11087002

[B47] Marti S. Dehaene S. (2017). Discrete and continuous mechanisms of temporal selection in rapid visual streams. *Nat. Commun.* 8:1955. 10.1038/s41467-017-02079-x 29208892 PMC5717232

[B48] McArthur G. Budd T. W. Michie P. T. (1999). The attentional blink and P300. *Neuroreport* 10 3691–3695. 10.1097/00001756-199911260-00042 10619668

[B49] McCarthy G. Puce A. Constable T. Krystal J. H. Gore J. C. Goldman-Rakic P. (1996). Activation of human prefrontal cortex during spatial and nonspatial working memory tasks measured by functional MRI. *Cereb. Cortex* 6 600–611. 10.1093/cercor/6.4.600 8670685

[B50] Melloni L. Molina C. Pena M. Torres D. Singer W. Rodriguez E. (2007). Synchronization of neural activity across cortical areas correlates with conscious perception. *J. Neurosci.* 27 2858–2865. 10.1523/JNEUROSCI.4623-06.2007 17360907 PMC6672558

[B51] Nakatani C. Ito J. Nikolaev A. R. Gong P. Leeuwen C. (2005). Phase synchronization analysis of EEG during attentional blink. *J. Cogn. Neuroence* 17 1969–1979. 10.1162/089892905775008706 16356332

[B52] Nieuwenstein M. R. (2006). Top-down controlled, delayed selection in the attentional blink. *J. Exp. Psychol. Hum. Percept. Perform.* 32 973–985. 10.1037/0096-1523.32.4.973 16846292

[B53] Nieuwenstein M. R. Chun M. M. van der Lubbe R. H. J. Hooge I. T. C. (2005). Delayed attentional engagement in the attentional blink. *J. Exp. Psychol. Hum. Percept. Perform.* 31 1463–1475. 10.1037/0096-1523.31.6.1463 16366802

[B54] Nobre A. C. Sebestyen G. N. Gitelman D. R. Mesulam M. M. Frackowiak R. S. J. Frith C. D. (1997). Functional localization of the system for visuospatial attention using positron emission tomography. *Brain* 120 515–533. 10.1093/brain/120.3.515 9126062

[B55] Nuwer M. R. Comi G. Emerson R. Fuglsang-Frederiksen A. Guerit J.-M. Hinrichs H. (1999). IFCN standards for digital recording of clinical EEG. The International Federation of Clinical Neurophysiology. *Electroencephalogr. Clin. Neurophysiol. Suppl.* 52 11–14. 10590972

[B56] Olivers C. N. L. Nieuwenhuis S. (2006). The beneficial effects of additional task load, positive affect, and instruction on the attentional blink. *J. Exp. Psychol. Hum. Percept. Perform.* 32 364–379. 10.1037/0096-1523.32.2.364 16634676

[B57] Quintana J. Fuster J. M. (1999). From perception to action: temporal integrative functions of prefrontal and parietal neurons. *Cereb. Cortex* 9 213–221. 10.1093/cercor/9.3.213 10355901

[B58] Rashid B. Arbabshirani M. R. Damaraju E. Cetin M. S. Miller R. Pearlson G. D. (2016). Classification of schizophrenia and bipolar patients using static and dynamic resting-state fMRI brain connectivity. *NeuroImage* 134 645–657. 10.1016/j.neuroimage.2016.04.051 27118088 PMC4912868

[B59] Raymond J. E. Shapiro K. L. Arnell K. M. (1992). Temporary suppression of visual processing in an RSVP task: an attentional blink? *J. Exp. Psychol. Hum. Percept. Perform.* 18 849–860. 10.1037/0096-1523.18.3.849 1500880

[B60] Sergent C. Baillet S. Dehaene S. (2005). Timing of the brain events underlying access to consciousness during the attentional blink. *Nat. Neurosci.* 8 1391–1400. 10.1038/nn1549 16158062

[B61] Shapiro K. L. Raymond J. E. Arnell K. M. (1994). Attention to visual pattern information produces the attentional blink in rapid serial visual presentation. *J. Exp. Psychol. Hum. Percept. Perform.* 20 357–371. 10.1037/0096-1523.20.2.357 8189198

[B62] Shapiro K. Schmitz F. Martens S. Hommel B. Schnitzler A. (2006). Resource sharing in the attentional blink. *NeuroReport* 17 163–166. 10.1097/01.wnr.0000195670.37892.1a 16407764

[B63] Singer W. (1999). Neuronal synchrony: a versatile code for the definition of relations? *Neuron* 24 49–65. 10.1016/S0896-6273(00)80821-1 10677026

[B64] Slagter H. A. Lutz A. Greischar L. L. Francis A. D. Nieuwenhuis S. Davis J. M. (2007). Mental training affects distribution of limited brain resources. *PLoS Biol.* 5 1228–1235. 10.1371/journal.pbio.0050138 17488185 PMC1865565

[B65] Squarcina L. Castellani U. Bellani M. Perlini C. Lasalvia A. Dusi N. (2017). Classification of first-episode psychosis in a large cohort of patients using support vector machine and multiple kernel learning techniques. *NeuroImage* 145 238–245. 10.1016/j.neuroimage.2015.12.007 26690803

[B66] Steinu A. Von Sarnthein J. (2000). Different frequencies for different scales of cortical integration: from local gamma to long range alpha/theta synchronization. *Int. J. Psychophysiol.* 38 301–313. 10.1016/S0167-8760(00)00172-0 11102669

[B67] Teufel C. Fletcher P. C. (2020). Forms of prediction in the nervous system. *Nat. Rev. Neurosci.* 21 231–242. 10.1038/s41583-020-0275-5 32221470

[B68] Uhlhaas P. J. Pipa G. Lima B. Melloni L. Neuenschwander S. Nikolic D. (2009). Neural synchrony in cortical networks: history, concept and current status. *Front. Integr. Neurosci.* 3 1–19. 10.3389/neuro.07.017.2009 19668703 PMC2723047

[B69] Varela F. Lachaux J. Rodriguez E. Martinerie J. (2001). The brainweb: phase synchronization and large-scale integration. *Nat. Rev. Neurosci.* 2 229–239. 10.1038/35067550 11283746

[B70] Vogel E. K. Luck S. J. (2002). Delayed working memory consolidation during the attentional blink. *Psychon. Bull. Rev.* 9 739–743. 10.3758/BF03196329 12613677

[B71] Vogel E. K. Luck S. J. Shapiro K. L. (1998). Electrophysiological evidence for a postperceptual locus of suppression during the attentional blink. *J. Exp. Psychol. Hum. Percept. Perform.* 24 1656–1674. 10.1037/0096-1523.24.6.1656 9861716

